# Genomewide Mapping of Selection Signatures and Genes for Extreme Feather Pecking in Two Divergently Selected Laying Hen Lines

**DOI:** 10.3390/ani10020262

**Published:** 2020-02-06

**Authors:** Hanna Iffland, Robin Wellmann, Markus Schmid, Siegfried Preuß, Jens Tetens, Werner Bessei, Jörn Bennewitz

**Affiliations:** 1 Institute of Animal Science, University of Hohenheim, Garbenstraße 17, 70593 Stuttgart, Germany; r.wellmann@uni-hohenheim.de (R.W.); markus_schmid@uni-hohenheim.de (M.S.); preuss@uni-hohenheim.de (S.P.); bessei@uni-hohenheim.de (W.B.); j.bennewitz@uni-hohenheim.de (J.B.); 2 Department of Animal Science, University of Göttingen, Burckhardtweg 2, 37077 Göttingen, Germany; jens.tetens@uni-goettingen.de

**Keywords:** laying hens, selection signatures, extreme feather pecking, divergent selection, QTL

## Abstract

**Simple Summary:**

Feather pecking is a behavior frequently occurring in commercial layer flocks. It often leads to skin injuries and cannibalism. Besides economic losses, severe animal welfare problems cannot be ignored. Previous research has shown that the trait is heritable. Thus breeding against feather pecking is possible, but phenotyping in a commercial environment is economically unfeasible at the moment because of the lack of proper techniques. Therefore, understanding the genetic background of the trait is mandatory to establish a genomic breeding program. This would require genotypic information of the hens, which is feasible under practical conditions. In the present study, we used different methods to identify regions in the genome that influence feather pecking and extreme feather pecking. We found one trait associated with the genomic region. The use of genotypic information from this region in terms of selection against the undesired behavior may help to improve animal welfare in layer flocks.

**Abstract:**

Feather pecking (FP) is a longstanding serious problem in commercial flocks of laying hens. It is a highly polygenic trait and the genetic background is still not completely understood. In order to find genomic regions influencing FP, selection signatures between laying hen lines divergently selected for high and low feather pecking were mapped using the intra-population iHS and the inter-population F_ST_ approach. In addition, the existence of an extreme subgroup of FP hens (EFP) across both selected lines has been demonstrated by fitting a mixture of negative binomial distributions to the data and calculating the posterior probability of belonging to the extreme subgroup (pEFP) for each hen. A genomewide association study (GWAS) was performed for the traits pEFP and FP delivered (FPD) with a subsequent post GWAS analysis. Mapping of selection signatures revealed no clear regions under selection. GWAS revealed a region on Chromosome 1, where the existence of a QTL influencing FP is likely. The candidate genes found in this region are a part of the GABAergic system, which has already been linked to FP in previous studies. Despite the polygenic nature of FP, selection on these candidate genes may reduce FP.

## 1. Introduction

Feather pecking is a long known and still existing problem in commercial flocks of laying hens. It causes feather damages and skin lesions often resulting in cannibalism, which is an animal welfare issue and leads to economic losses. For years it has been a topic of intensive research and many aspects influencing feather pecking have already been revealed like stocking density, light intensity, nutrition, or litter [1]. Besides these environmental factors, genetic factors were shown to influence the occurrence of feather pecking as well. Low to medium heritabilities were frequently found [2,3] indicating the possibility to breed for this trait which has also been proven in several selection experiments [4]. A number of mapping studies revealed quantitative trait loci (QTL) and trait-associated genome-regions, but also highlighted the quantitative and complex nature of this trait [5,6], because the few significant QTL by far did not explain the total genetic variance.

One approach to identify candidate regions in the genome is to map selection signatures in selected lines. As selection increases the frequency of an advantageous allele, nearby variants in linkage disequilibrium (LD) with the superior allele will also increase in frequency, termed as “hitch-hiking” alleles. At the beginning of selection, this results in long haplotype segments that are surrounding the advantageous alleles. Their increase in frequency in the population is called a “selective sweep” which leads to a reduced genetic diversity in the vicinity of advantageous alleles [7,8]. Hence, selection pressure leads to a specific formation of selection signatures which can be detected with several inter- and intra-population methods. In this study, two lines selected for high (HFP) and low (LFP) feather pecking for 15 generations were used to map selection signatures using the intra-population haplotype-based integrated haplotype score (iHS) [9] and the inter-population SNP-based F_ST_ index [10,11]. Chromosomal regions with significant selection signatures point to the presence of QTL within these regions.

Feather pecking is not homogenous within groups of laying hens and several studies reported the presence of a subgroup of extreme feather peckers (EFP), i.e., showing an exceptional high severe feather pecking activity compared to group mates [12,13,14,15]. In a previous study [15], we detected a subgroup of extreme feather pecking hens within an F2-cross of the HFP and LFP lines mentioned above by fitting a mixture of two negative binomial distributions to feather pecking data. A proportion of 33% extreme feather pecking hens was found. We also showed that extreme feather pecking hens came more than twice as often into the motivation period for feather pecking and pecked about five times more feathers than the other hens when they were in the motivation period. This led to the conclusion that genetic strategies should focus on extreme feather pecking. Consequently, it is important to detect the individual extreme feather peckers and to analyze their genetic background. Using the results from the fit of the mixture of negative binomial distributions, the posterior probability of belonging to the distribution representing the extreme feather pecking subgroup was calculated for each hen. We used this probability (pEFP) as a new trait for extreme feather pecking and a genomewide association study revealed several significant SNPs for pEFP [15].

The aim of the present study was to map selection signatures in lines that were divergently selected for feather pecking. In a second step, we identified extreme feather pecking hens by fitting a mixture of two negative binomial distributions to feather pecking data and calculated the new trait pEFP. Finally, genomic regions associated with feather pecking were identified by conducting a GWAS for the traits feather pecks delivered (FPD) and pEFP. Those regions were further analyzed to reveal the putative candidate genes.

## 2. Materials and Methods

### 2.1. Experimental Design, Data Collection and Editing

Based on a founder line of a White Leghorn layer strain established in 1970 as a control population in the Scandinavian selection and cross-breeding experiment of Liljedahl et al. [16], divergent selection for HFP and LFP started in 1996 at the Danish Institute of Agricultural Sciences [17]. Breeding values for feather pecking behavior were estimated and used for selection in the HFP line and in the LFP line. After five generations, fertilized eggs were transferred to the experimental farm of the University of Hohenheim in Germany where the selection for HFP and LFP continued. Current data was generated by phenotyping hens of the HFP and LFP lines of the 15th generation, hatched in 2017. Rearing and husbandry conditions have not been changed since then and are briefly described in Bennewitz et al. [3].

Three hatches were produced in two week intervals. The hens of the first two hatches took part in the first experimental run at the age of 33 and 31 weeks, respectively and hatch three in the second run at the age of 32 weeks. One week before behavioral observations, the hens were marked with numbered plastic tags on their back for individual identification and then transferred to observation pens in a ratio of 1:1 of LFP to HFP hens and around 42 individuals per pen in at total seven pens in the first run.

Because of less total hens and a lower number of LFP in the second run, six groups each of about 40 hens were used with a ratio of 1:2 LFP to HFP. A 14 hours’ light program was provided by incandescent bulbs from 3 am to 5 pm. There was additional natural light through transparent plastic material at the upper part of the side walls. Depending on the fluctuation of the natural light, light intensity increased occasionally from 20 up to 2500 lux.

Observations were done in two sessions each day, starting at 10 am on four consecutive days. In order to ensure a balanced observation scheme, the number of observers corresponded with the number of observation pens (7 in the first and 6 in the second run). Each pen was observed by each observer on each day in 20 min sessions. The observers changed the compartments in a rotational system. This resulted in at total 560 min of observation time in the first run and 480 min in the second. To gain compatibility with data from an F2 design of these lines, observation time was standardized to 420 min [18]. FPD as well as feather pecks received were recorded in the morning sessions whereas aggression was recorded in the afternoon (not further analyzed in this study).

FPD was recorded as non-aggressive severe pecks or pulls directed to the plumage of group members with sometimes resulting in pulled out feathers and a recipient which tolerates or moves away [18,19]. A series of pecks delivered in a short sequence without the hen changing its behavior were recorded as a single occurrence and called a bout per bird (bpb). A number of 492 hens (270 HFP and 222 LFP) were phenotyped sufficiently.

For the GWAS, FPD was Box-Cox transformed to reduce the deviation of the distribution from a normal distribution. After adding 1 to the FPD recordings the following transformation was applied: $y_{ti}\;=\;\frac{\left(y_{i}^{-0.2}-1\right)}{-0.2}$, where *y_i_* is the number of bpb of each hen *i* and *y_ti_* is the transformed observation. The power parameter −0.2 was used according to Lutz et al. [20] and Su et al. [21] showing the best fit for feather pecking data.

Blood was collected from the hens to extract the DNA and to perform genotyping with the Illumina 60 K chicken Infinium iSelect chip. SNPs with a call frequency lower than 0.95 and a minor allele frequency of zero were filtered out. Additionally, SNPs located on the sex chromosomes as well as SNPs that were not allocated to a specific chromosome according to positional information of the chicken genome assembly GRCg6a were excluded. This filtering resulted in 29,020 SNPs and 494 hens (270 HFP and 219 LFP) with sufficient genotype information. Sporadic missing genotypes were imputed and the genotypes were phased with Beagle 5.0 [22]. The total amount of hens with sufficient phenotypic as well as genotypic data was 489.

The research protocol was approved by the German Ethical Commission of Animal Welfare of the Provincial Government of Baden-Wuerttemberg, Germany (code: HOH 35/15 PG, date of approval: 25 April 2017).

### 2.2. Statistical Analysis

#### 2.2.1. Multidimensional Scaling

In order to visualize the genetic distance between the divergently selected feather pecking lines, a multidimensional scaling was performed. In the first step, using R package “optiSel” [23], the segment-based kinship $f_{SEG}\;\left(i,j\right)$ between all pairs of individuals i,j was calculated. Each segment comprised at least 20 markers and was at least 3.3 cM long.

The 20 markers per segment are considered to be enough to ensure that two segments with identical marker alleles are not identical by chance [24]. In accordance with Browning [25], the minimum length of a segment was chosen as 3.3 = $\frac{100}{2g}$ cM, where *g* denotes the number of generations after the base population has been established, which is 15 in our case. It needs to be ensured that the number of SNPs that remained in the dataset after filtering is enough to deliver segments which are sufficiently long. This can be seen as follows. Under the assumption of equally spaced SNPs, $0.167\;=\;\frac{100}{40\;g}$ cM is the maximum allowed marker distance between adjacent SNPs if segments cover at least 20 markers [26]. This is more than four times larger than the actual average marker distance, which is $\frac{10.4\;\cdot 100\;}{29020}\;=\;0.035$ cM, where 10.4 M is the length of the chicken genome. The shortest detectable segment is thus 20·0.035 = 0.7 cM long, which is considerably smaller than the minimum segment length, which was set as 3.3 cM. Even under the knowledge that the SNPs are not equally spaced, more than four times as many seem to be a sufficient number of SNPs.

The kinship between individuals i,j was calculated as

(1) $$f\left(i,j\right)\;=\;0.02+0.98f_{SEG}\left(i,j\right),$$

where $f_{SEG}\left(i,j\right)$ is the segment-based kinship between individuals i and j, and 0.02 is the ancestral kinship that is assumed to be not covered by the markers. The kinship, which is a value between 0 and 1, was then mapped to the positive real numbers and converted into a dissimilarity measure with function

(2) $$Dissimilarity\left(i,j\right)\;=\;\log{\left(f\left(i,j\right)\right)}^{2}.$$

The individuals were arranged on a two-dimensional plane such that their distances correspond to their genetic dissimilarities as good as possible by performing multidimensional scaling with R package “smacof” [27].

#### 2.2.2. Mapping Selection Signatures

Selection signatures within each line were mapped with the iHS statistic of R package “rehh” [28,29]. The test for a single population compares the average lengths of haplotype segments around a focal SNP s that carry the ancestral allele A with those that carry the derived allele D. For each SNP, the allele with the highest average frequency over both populations was set as ancestral and the other as derived. A selection signature is detected if both average segment lengths differ significantly from each other. The average length of haplotype segments that carry a given allele a∈{A,D} at position s is estimated by the integrated extended haplotype homozygosity $IHH_{s}^{a}$ as follows. Two haplotypes are assumed to be chosen at random without replacement from the population from all haplotypes that carry the a-allele, and the proportion $EHH_{s,t}^{a}$ of cases is computed in which they are identical between positions s and t. This value decreases from 1 to 0 when SNP t moves away from the focal SNP s. The average haplotype segment length $IHH_{s}^{a}$ is then estimated as the integral of $EHH_{s,t}^{a}$ over all SNP *t* with $EHH_{s,t}^{a}\;>\;0.05$. The test statistic is a monotone function of

(3) $$\mathrm{unstandardized}\;iHS_{\mathrm{pop}i}\left(s\right)\;=\;\ln\left(\frac{IHH_{s}^{A}}{IHH_{s}^{D}}\right).$$

If the value is smaller than 0, then haplotypes that carry the derived allele are on average longer than the haplotypes that carry the ancestral allele, so it might be expected that the derived allele has swept up in frequency. A large positive value, however, can also indicate a selective sweep. This is the case when ancestral alleles hitchhike with the selected site. Therefore, a two-sided test was carried out. The standardized iHS value was used as the test statistic. The standardization was done conditionally on the allele frequency, which removed the effect of the allele frequency on the distribution of the test statistic. The “rehh” package assumes that the standardized iHS value has a normal distribution [9]. For this test, only SNPs with a minor allele frequency (MAF) larger than 0.01 within each line were considered, which resulted in 22,425 SNPs for the HFP and 23,084 SNPs for the LFP line. Both lines had 16,766 of these SNPs in common.

In order to increase the power of the iHS test, the test was also carried out for both populations simultaneously. First, the standardized iHS values were calculated separately for the two divergently selected lines. The standardized difference of both iHS values was then used as the test statistic, i.e.,

(4) $$\mathrm{combined}\;iHS\left(s\right)\;=\;\frac{iHS_{\mathrm{pop}1}\left(s\right)-iHS_{\mathrm{pop}2}\left(s\right)}{mad\left(iHS_{\mathrm{pop}1}-iHS_{\mathrm{pop}2}\right)}.$$

Thereby, the mad is the scaled median absolute deviation, which is a robust estimate for the standard deviation of a normal distribution. The test statistic has therefore a standard normal distribution under $H_{0}$. The rationale behind this approach is that signatures from selective sweeps in both lines that result from selection in the same direction tend to cancel each other, whereas signatures from selective sweeps that result from divergent selection increase in magnitude. Since the lines are divergently selected for feather pecking, it is likely that the resulting selection signatures result from selection on feather pecking. As large negative and large positive values both indicate a selective sweep, the p-values for the combined iHS test were calculated as

(5) $$p_{iHS}\;=\;2\Phi \left(-\left|\mathrm{combined}\;iHS\left(s\right)\right|\right),$$

where Φ() is the distribution function of the standard normal distribution.

Selection signatures between the HFP and LFP lines were mapped using the genetic differentiation index *F*_*ST*_. Similar allele frequencies in the two subpopulations are represented by small *F*_*ST*_ values whereas different allele frequencies lead to large *F*_*ST*_ values and thus indicate regions under selection [10]. The *F*_*ST*_ index was already calculated in Grams et al. [30] for data of hens of the 11th generation of the two feather pecking lines. The same approach is used in the current study and hence in the following only described briefly. *F*_*ST*_ indices were computed using Equation (8) of Weir and Cockerham [11]:

(6) $$F_{ST}=\frac{\sigma_{p}^{2}}{\bar{p}\left(1-\bar{p}\right)},.\;$$

where $\sigma_{p}^{2}$ is the variance of the allele frequency across the two lines and is estimated as $\sigma_{p}^{2}\;=\;\left(\bar{p^{2}}\right)-\left({\bar{p}}^{2}\right)$, where $\bar{p^{2}}$ is the mean of the squared allele frequencies in the two lines. $\bar{p}$ is the mean allele frequency for the two lines.

To account for differences in allele frequencies because of genetic drift, a statistical test was developed by Grams et al. [30]. The distribution of the *F*_*ST*_-values under the null-hypothesis that an allele is neutral was obtained by simulation. Each allele was given a starting allele frequency of 0.5 in the base population. The allele frequencies for the two populations were simulated for 15 generations. For the first 11 generations, the effective population size (Ne) was the same as in Grams et al. [30]. From generation 12 to 15, the Ne was 40 because of a wider selection of breeding animals in these generations. The simulation was repeated 100,000 times resulting in 100,000 *F*_*ST*_ values. This revealed the distribution of *F*_*ST*_ indices under the null hypothesis of genetic drift but without selection. Finally, the *p*-value for each real SNP (*p*_*nominal*_) was computed as the proportion of simulated SNPs that showed a greater *F*_*ST*_ index than the SNP under consideration. *F*_*ST*_ computations were performed using an own written R script.

The correction for multiple testing was done by applying a Bonferroni correction as $p_{genomewide}\;=\;1-{\left(1-p_{nominal}\right)}^{\#SNP}$, where the number of SNPs was 29,020 and the significance level *p*_*genomewide*_ was set to ≤ 0.05. The other two additional levels of significance were set to *p*_*nominal*_ ≤ 5 × 10^−4^ and *p*_*nominal*_ ≤ 5 × 10^−5^ as well due to the very conservative approach of the Bonferroni correction. False discovery rates (FDR) for every *p*_*nominal*_ value were calculated with the R package “qvalue” [31] to estimate the number of false positives among the significant SNPs.

In a final step, a meta-analysis was performed by combining the *p*-values of the combined iHS test and the *F*_*ST*_ test. This was done as follows using Fisher’s combined probability test [32]:

(7) $$\chi_{2k}^{2}\sim -2\overset{k}{\underset{i\;=\;1}{\sum }}\ln\left(p_{i}\right),$$

where k is the number of studies being combined (k = 2 in this study) and $p_{i}$ is the *p*-value for the $i^{th}$ hypothesis test. The significance level that was used for the meta-analysis was the same as for the iHS statistics.

#### 2.2.3. Detecting Extreme Feather Peckers

Iffland et al. [15] introduced a novel method to detect extreme feather peckers in a group of laying hens, which was applied to the two lines jointly as described briefly in the following. It is assumed that each hen belongs to one of two subgroups, which are the extreme feather pecking (EFP) and the normal feather pecking (FP) subgroup. Hence, the density of the number of bouts per bird $N_{bpb}$ is

(8) $$f\left(N_{bpb}\right)=\;\pi \;f_{EFP}\left(N_{bpb}\right)+\left(1-\pi \right)f_{FP}\left(N_{bpb}\right),$$

where π is the proportion of EFP hens in the population. Here, $f_{EFP}\left(N_{bpb}\right)$ is the density of $N_{bpb}$ in the EFP subgroup, and $f_{FP}\left(N_{bpb}\right)$ is the density of $N_{bpb}$ in the FP subgroup. It is assumed that $N_{bpb}$ has a negative binomial distribution within each subgroup. The distributions have parameters $\mu_{EFP},\sigma_{EFP}$ and $\mu_{\mathrm{FP}},\sigma_{\mathrm{FP}}$, respectively, where $\mu_{j}$ is the mean of $N_{bpb}$ for subgroup *j* (*j* = EFP or FP) and $\sigma_{j}$ is the standard deviation. The parameters were estimated with R package “mixdist” [33].

If a hen belongs to subgroup j, then its number of bouts can be written as $N_{bpb}\;=\;\overset{N_{j}}{\underset{k=1}{\sum }}X_{kj}$, where $N_{j}$ is the number of times the hen came into the pecking motivation period (i.e., time period with motivation for feather pecking), and $X_{kj}$ is the number of pecks when the hen came into the pecking motivation period for the *k*-th time. These values can be estimated from the results of the fit of the mixture of negative binomial distributions as follows [15]

(9) $$E\left(N_{j}\right)\;=\;\lambda_{j}\;=\;-\frac{\mu_{j}^{2}}{\sigma_{j}^{2}-\mu_{j}}\ln\left(\frac{\mu_{j}}{\sigma_{j}^{2}}\right),\;\mathrm{and}\;E\left(X_{kj}\right)\;=\;\frac{\mu_{j}}{\lambda_{j}}.$$

Hence, the separation into pecking motivation periods and number of pecks within pecking motivation periods was not part of the applied ethogram, but is our interpretation of the parameters of the mixture distributions. In a second step, using the estimated parameters of the fit of the mixture distributions, the posterior probability for each hen to belong to the EFP subgroup (pEFP) is calculated as follows:

(10) $$pEFP\;=\;P(j\;=\;EFP\;|N_{bpb})\;=\frac{\pi f_{EFP}\left(N_{bpb}\right)}{f\left(N_{bpb}\right)},$$

where $P(j\;=\;EFP|N_{bpb})$ is the posterior probability for a hen with $N_{bpb}$ bpb to belong to the EFP subgroup. Finally, the new trait pEFP was dichotomized at a threshold of 0.5 for further analysis.

#### 2.2.4. GWAS and Estimations of Genetic Parameters

To analyze the genetic background of pEFP and FPD, a single-marker GWAS was carried out using the software GCTA [34]. The chromosome with the candidate SNP was excluded from calculating the genetic relationship matrix *G*. For each of the two traits, the following model was applied:

(11) y = Xb+Wu+g+e,

where *y* is a vector of observations of the corresponding trait, *b* is a vector containing the fixed effect of the line as well as a fixed combinational effect consisting of the experimental run and pen. *X* is the corresponding design matrix, *u* is a vector of the fixed SNP effects to be tested, and *W* is the standardized genotype matrix. The random vector *g* contains the additive animal effects with $g\sim N\left(0,\sigma_{g}^{2}G\right)$, where *G* is the genomic relationship matrix. The vector *e* residual effects has distribution $e\sim N\left(0,\sigma_{e}^{2}I\right)$ with *I* being the identity matrix. For GWAS computation, the minor allele frequency (MAF) was set to 0.01, which resulted in 28,525 remaining SNPs.

The variance components were also estimated with GCTA [34], where the model was reduced to: y = Xb+g+e.

A prevalence of 0.24 was specified for pEFP to transform the heritability estimated on the observed scale within a case-control threshold model on the liability scale. The prevalence was estimated from the data.

#### 2.2.5. Post GWAS Analysis

In regions of the genome where QTL are likely according to the GWAS results of pEFP and FPD, screening for positional candidate genes was done going from significant Peak-SNP 1.5 Mb up- and downstream using the NCBI (National Center for Biotechnology Information) genome data viewer. The positional candidate genes were then analyzed with the database for annotation, visualization and integrated discovery (DAVID) version 6.8 [35,36] to perform a functional annotation clustering and thus to identify enriched annotation terms. For clustering, a similarity threshold of 0.85, an EASE score of 0.1 and an enrichment score of ≥1.3 (*p* ≤ 0.05) were used as stated out in Huang et al. [36].

## 3. Results

### 3.1. Multidimensional Scaling

**Figure 1:**
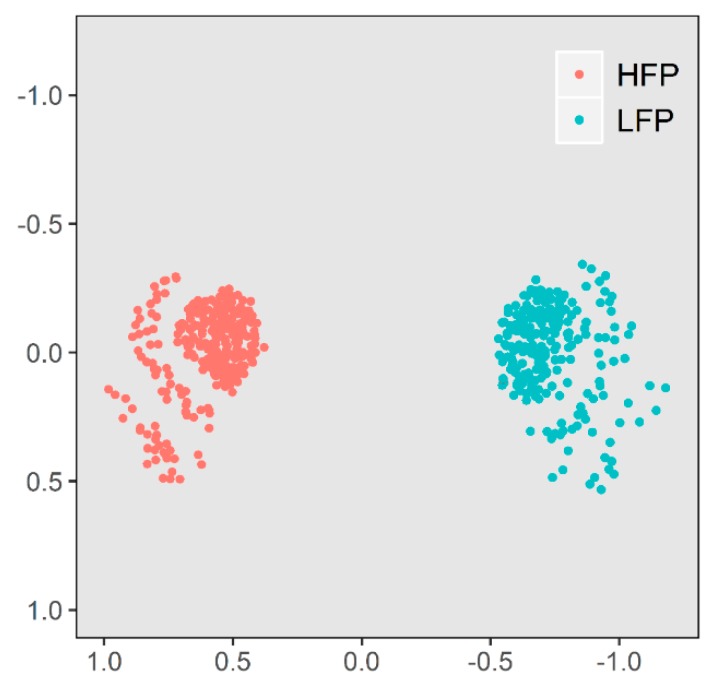
Multidimensional scaling of the high (HFP, n = 272) and low (LFP, n = 222) feather pecking lines. The distance to each other visualize their genetic distance.

The multidimensional scaling plot can be seen in Figure 1. HFP and LFP lines are clearly separated from each other with both having two crescent-shaped substructures in their clusters.

**Figure 2:**
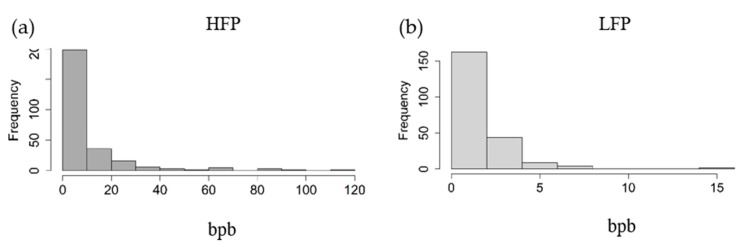
Histogram of feather pecks delivered in bouts per bird (bpb) for (**a**) the high (HFP, n = 270) and (**b**) low (LFP, n = 222) feather pecking lines. Note the different scales used for clarity.

Figure 2 shows the histograms of bpb of each of the two lines. Hens of the HFP line are pecking considerably more often, compared to the hens of the LFP line.

### 3.2. Selection Signatures

**Figure 3:**
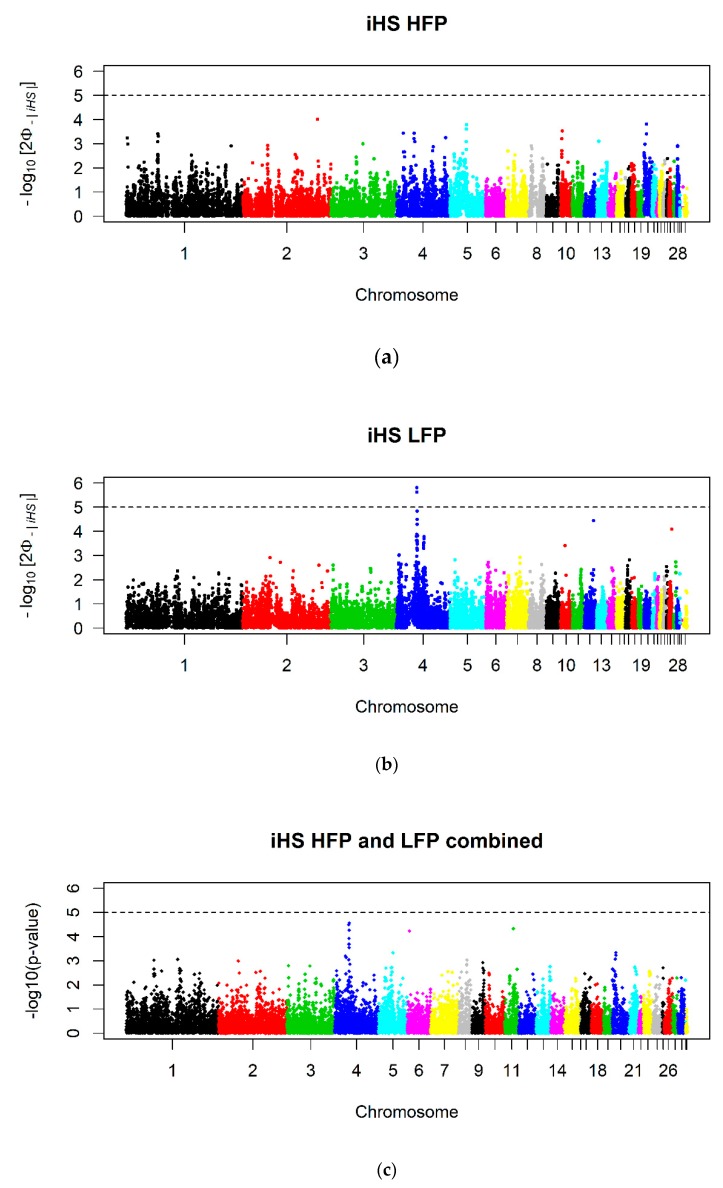
*P*-values of the integrated haplotype score (iHS) for (**a**) the high feather pecking line (HFP, n_SNP_ = 22,425), (**b**) the low feather pecking line (LFP, n_SNP_ = 23,084) and (**c**) their combination (n_SNP_ = 16,766). The dashed lines indicate the nominal level of significance *p* ≤ 10^−5^.

For LFP, the iHS approach revealed two nominal significant (*p* ≤ 10^−5^) SNPs on Chromosome 4 (Figure 3). No significant SNPs and thus no selection signatures could be found for HFP or for the combination of both lines (Figure 3).

**Figure 4:**
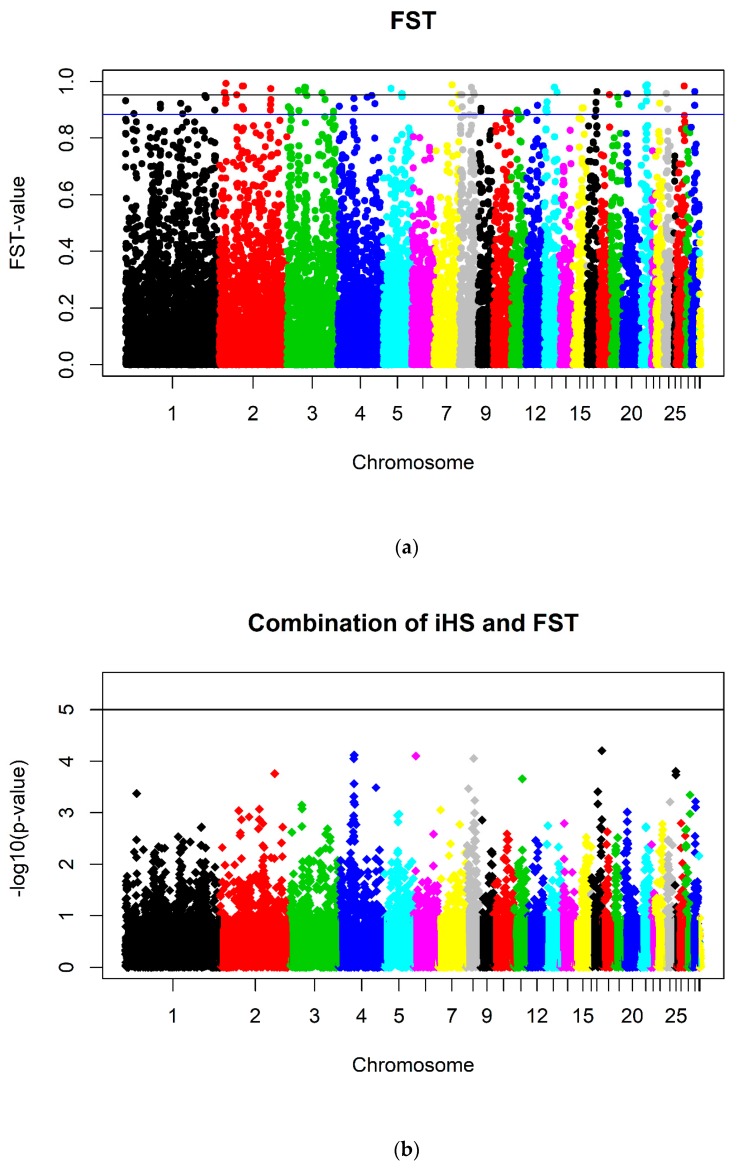
(**a**) Manhattan plot of F_ST_-indices (n_SNP_ = 29,020). The top line indicates the nominal level of significance *p*_*nominal*_ ≤ 5 × 10^−5^ and the bottom line the nominal level of significance *p*_*nominal*_ ≤ 5 × 10^−4^. (**b**) Manhattan plot of the −log_10_ *p*-values of the combination of the combined iHS *p*-values and the F_ST_ p-values (n_SNP_ = 16,766). The line indicates the nominal level of significance *p* ≤ 10^−5^.

Results of F_ST_ statistics are shown in Figure 4. A number of 57 SNPs on 13 chromosomes reached significance (*p*_*nominal*_ ≤ 5 × 10^−5^) with an FDR of 0.021. With the relaxed level of significance (*p*_*nominal*_ ≤ 5 × 10^−4^), another 92 SNPs on in total 22 chromosomes reached significance (FDR 0.056). The most significant SNPs (*p*_*nominal*_ ≤ 5 × 10^−4^) were found (in descending order) on Chromosomes 2, 3, 1, 8 and 11. No genomewide significant SNPs could be revealed because SNPs did not reach genomewide significance.

The meta-analysis of the combination of the *p*-values of the combined iHS statistic and the F_ST_ indices is plotted in Figure 4. No SNPs reached significance.

### 3.3. Extreme Feather Peckers

**Figure 5:**
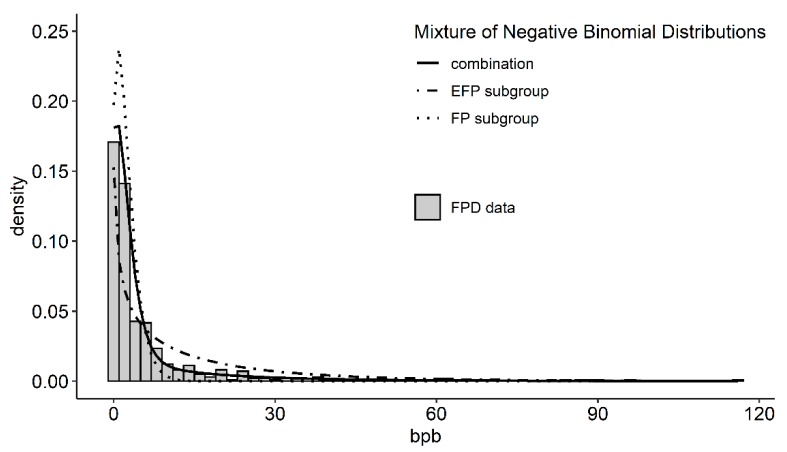
Mixture of negative binomial distribution (black lines) fitted to the feather pecking data (grey bars) in bouts per bird (bpb) of the high and low feather pecking hens (n = 492). The dotted line represents the fit of the feather pecking (FP) subgroup, the dot-dashed line represents the extreme feather pecking (EFP) subgroup and the solid line represents the combination of both and visualizes the good of fit of the mixture distributions.

In Figure 5, the combination of the feather pecking data of both lines is shown with the mixture of two negative binomial distributions fitted to it to reveal the FP and EFP subgroups. Hens in the FP subgroup pecked feathers on average ${\hat{\mu }}_{FP}\;=$ 2.26 (SE 0.33) times during observation and made up $1-\hat{\pi }\;=\;\;$63% of the whole experimental population. Additionally, they came on average $\lambda_{FP}\;=\;\;$1.62 times into pecking motivation period, in which case they pecked feathers on average $E(X_{ikFP})\;=\;\;$1.65 times. In contrast, hens from the EFP subgroup pecked feathers on average ${\hat{\mu }}_{EFP}\;=$ 13.8 (SE 4.25) times during observation and made up $\hat{\pi }\;=\;$37% of the experimental population. They came on average $\lambda_{EFP}\;=\;$1.88 times into pecking motivation period in which case they pecked feathers on average $E(X_{ikEFP})\;=\;$7.34 times.

### 3.4. GWAS and Estimations of Genetic Parameters

**Figure 6:**
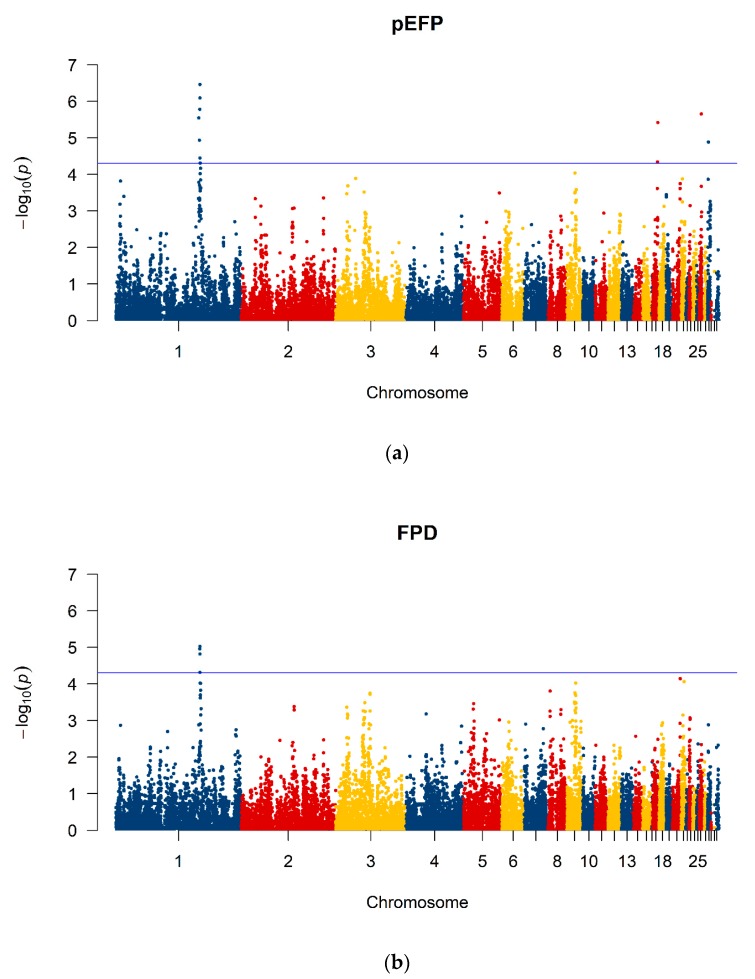
Manhattan plots of the −log_10_ *p*-values for association of SNPs (n_SNP_ = 28,525) for (**a**) the posterior probability of extreme feather pecking (pEFP) and (**b**) feather pecks delivered (FPD). The line indicates the nominal level of significance *p* ≤ 5 × 10^−5^.

GWAS revealed four nominal significant (*p* ≤ 5 × 10^−5^) SNPs on Chromosome 1 for FPD (Figure 6). For pEFP, seven significant SNPs on Chromosome 1 were found including the same four mentioned above for FPD, two on Chromosome 17 and one on Chromosome 26 and 28 (Figure 6), respectively. The full list of nominal significant SNPs can be seen in Table 1.

The estimated variance components and heritabilities for the two traits FPD and pEFP are shown in Table 2. For both traits, the heritabilities are in a medium range with 0.20 (SE 0.08) for FPD on the observed scale and 0.26 (SE 0.14) for the binary trait pEFP on the liability scale.

### 3.5. Post GWAS Analysis

Genes were screened 1.5 Mb up- and downstream from the significant peak SNP Gga_rs14888608 of the GWAS analyses for pEFP and FPD on Chromosome 1 (Table 1). This resulted in the specific window of 131,289,468 bp-134,289,468 bp. Fifty-two positional candidate genes were found in this window and input to DAVID which itself identified 44 of these genes and used them for functional annotation clustering. One significant cluster was identified and revealed 15 significant enriched terms (Table 3). The terms are all related to the same three genes: GABRA5, GABRB3, and GABRG3. In three terms, two additional genes are involved, i.e., CNGA3 and RP2.

## 4. Discussion

As shown in Figure 1, two crescent-shaped structures are notable in both feather pecking lines which are based on genetic similarities within the lines. An explanation leading to the subgroups might be the fact that for some generations, the hens were mated in 10 half-sib families within each line. Nevertheless, two clear groups of HFP and LFP hens are notable visualizing the genetic difference between the two lines after 15 generations of separate breeding. The phenotypic differences between the two lines in the 15th generation can be seen in Figure 2. In Piepho et al. [14], feather pecking data of the 5th to the 11th generation of both lines is shown with the same phenotypic difference over these seven generations. Hence, the two feather pecking subpopulations are both phenotypically and genotypically distinguishable, but there was little selection response in the last generations.

Despite of the lack of recent selection response, one might expect to find selection signatures in both lines. Mapping of selection signatures in each line using the haplotype-based iHS approach revealed only two significant SNPs on Chromosome 4 for the LFP line and no significant SNPs for the HFP line. After combination of both statistics to increase the power of the test, there were no significant SNPs anymore. One explanation might be that the selection response in the first generations did not lead to detectable selective sweeps because of the low effective sizes the lines have had in the subsequent generations.

The results of the SNP-based F_ST_ statistic did not reveal any genomewide significant SNPs because the lines were separated for many generations and had a moderately low effective size, so SNPs could be divergently fixed by chance. The SNPs with very high F_ST_ values were equally distributed over the whole genome. The mean F_ST_ was 0.16. According to Akey et al. [37], this specific pattern of equally distributed significant SNPs is a sign of genetic drift and not selection. Selection, irrespective whether natural or artificial, would be more locus specific and thus lead to single significant peaks. The effective population size in each of the two feather pecking lines over the last 15 generations was on average only 35. A huge impact of drift is thus likely leading to differentiation between and uniformity within the subpopulations which in turn leads to huge allele frequency differences and thus great F_ST_ indices.

Nevertheless, Grams et al. [30] mapped selection signatures in the 11th generation of these lines using the F_ST_ index as well and found 13 clusters harboring significant SNPs on eight chromosomes with a concentration of significant SNPs (in descending order) on Chromosomes 4 and 3 using a sliding window approach. In the current study as well, Chromosome 3 harbored the second most significant SNPs. The mean F_ST_ index in Grams et al. [30] over all SNPs with 0.15 is slightly smaller showing that the allele frequency differences have increased further over the last four generations. The FDR for significant SNPs were also low. In contrast to Grams et al. [30], in the current study no genomewide significant SNPs were found and the number of significant SNPs in total was also lower with 342 in Grams et al. [30] versus 206 in this study. This might be due to the six times higher number of animals used in the current study.

After combining the p-values of the F_ST_ indices with the p-values of the combined iHS statistics in the meta-analysis, no more selection signatures reaching nominal significance could be found. A reason why no selection signatures could be detected might be that feather pecking is a highly polygenic trait influenced by many genes with small effects. During selection, selection pressure is distributed on those many genes leading to a slow accumulation of low to medium allele frequencies. Hence, there might be divergent selection but it is not detectable for us with the current study design (i.e., amount of animals, SNP density, applied methods).

Another reason for the lack of selection signatures might be as follows. At the beginning of the divergent selection, breeding was successful and the two lines differed recognizably from each other. Over the past generations, the lines remained divergent in the trait because it was still the basis of selection but no more breeding progress could be gained and thus only the status quo was maintained [30] (Figure 1). A threshold exists for both directions of the trait feather pecking. Low feather pecking cannot go below zero and extremely high feather pecking hens cannot be kept in their groups anymore due to their harmful behavior.

The fit of the mixture distributions revealed the existence of FP and EFP subgroups. The results are confirmed by a previous study where the mixture distributions were fitted to F2 cross FPD data [15]. The proportion of EFPs in the population of the F2 cross was 34% and thus slightly smaller than in the current study but they pecked feathers on average 2.7 times more often. Hens in the F2 cross came also more often in the pecking motivation period where they pecked more feathers as hens of the EFP subgroup in this study. The results of the FP subgroup are nearly the same in both studies.

Significantly associated SNPs for both traits with highly significant *p*-values for pEFP were found in one region on Chromosome 1. This indicates the presence of a QTL. Hence, screening of positional candidate genes was done in this window. The terms in the significant cluster of the functional annotation clustering are linked to neurotransmitter-gated ion-channels, particularly gamma-aminobutyric acid receptor A (GABA_A_). These receptors are made up of five subunits with several isoforms, which can be divided into the following classes: α (1–6), β (1–4), γ (1–3), δ, ε, θ, π, and ρ (1–3) [38]. The three candidate genes found in this study, which are related to all terms, are encoding for the subunits α5 (GABRA5), β3 (GABRB3), and γ3 (GABRBG3). GABA_A_ receptors are mainly located in the central nervous system. They act as inhibitory ion channels representing an important antagonist to excitatory forces regarding the transmission of axon potentials and thus neuronal activity in the brain [39]. These facts lead to the hypothesis that a mutation in the candidate genes cause a malfunction of the GABA_A_ receptors resulting in a loss of inhibitory processes for feather pecking. Further research is needed to get more detailed information on the role of GABA_A_ in this regard, for example via eQTL studies. Iffland et al. [15] already assumed that EFP hens might miss a regulatory factor preventing downregulation of extreme feather pecking. In a study by Poshivalov [40] it has been shown that mice that were kept isolated over 12 weeks became aggressive and antisocial. After application of a GABA agonist or an irreversible inhibitor of a GABA degrading enzyme aggressiveness decreased and sociability increased. This exemplifies the regulatory effect of GABA and the GABA_A_ receptors on behavior patterns. Bennewitz et al. [3] reported a positive genetic correlation between FPD and aggression in an F2 cross of the lines selected for high and low feather pecking. This leads to the suggestion that GABA may also influence social behavior in chickens. Brinker et al. [41] found a GABAergic system related candidate gene (GABBR2, Chromosome 2) for direct genetic effects for survival time which is linked to cannibalism in crossbred laying hens. Lutz et al. [6] found a candidate gene (SLC12A9) on Chromosome 9 for FPD in a study of a large F2 cross of the HFP and LFP lines. The SCL12 gene family plays a role in the GABAergic system as well. They also linked the serotonergic system to their findings because serotonin mediated by 5-HT_2_ receptors inhibits GABA_A_ receptor currents [42]. As reviewed by de Haas and van der Eijk [43] it was repeatedly shown that the serotonergic system can be genetically linked to feather pecking. Flisikowski et al. [44] identified serotonergic related genes (DRD4 and DEAF1) on Chromosome 5 to influence feather pecking behavior.

Direct observations by several observers for measuring FP behavior, as was used in this study, leads to some limitations. Although our observers were trained to differentiate between severe FP, gentle FP and aggressive pecking, a bias due to subjectivity cannot be completely excluded. In order to minimize this, we used as many observers as we had pens and hence all groups were observed by all observers at the same day in a rotational scheme. Thus, an inter-observer reliability was not calculated. The calculation of the intra-observer reliability requires video-records in order to provide the same feather pecking pattern repeatedly to the same observer. This technique, however, was not applicable in our study because of the relatively large groups with about 40 individually tagged hens. As reviewed by Ellen et al. [45], it could already be shown in the PhenoLab project that ultra-wideband as well as video tracking of hens of another HFP and LFP line explored differences in activity of both lines with an accuracy of up to 85% compared to the human observer [46]. It was also possible to detect individual FP hens due to their increased activity levels compared to the victims [46]. The use of this technology in the future is promising and would lead to more objectivity in measuring FP behavior.

## 5. Conclusions

Mapping of selection signatures in lines of laying hens divergently selected for feather pecking behavior revealed no clear regions under selection indicating that they are either not detectable with the current study design because of the polygenic nature of the trait or that there are no selection signatures because of the lack of stringent selection response over the last generations. A GWAS for the traits feather pecks delivered and the posterior probability of a hen belonging to the extreme feather peckers revealed a region on Chromosome 1 where the existence of QTL influencing the feather pecking phenomenon is likely. The candidate genes found in this region are a part of the GABAergic system which is related to the serotonergic system. Both systems were frequently linked to feather pecking which is confirmed by the current study as well. The candidate genes found in the present study may play an important role in the occurrence of the phenomenon. However, feather pecking is a quantitative trait influenced by many genes with more or less small effects.

## Figures and Tables

**Table 1:** Significant SNPs with *p* ≤ 5 × 10^−5^ from GWAS.

^1^ Trait	^2^ Chr	SNP	Position (bp)	−Log_10_ (p)
FPD	1	**GGaluGA044500**	132,686,520	4.96
	1	**Gga_rs14888608**	132,789,468	5.03
	1	**GGaluGA044531**	132,792,863	4.82
	1	**Gga_rs13940234**	132,960,547	4.32
pEFP	1	Gga_rs13938103	131,055,669	5.55
	1	Gga_rs14887858	132,015,352	4.94
	1	**GGaluGA044500**	132,686,520	5.78
	1	**Gga_rs14888608**	132,789,468	6.46
	1	**GGaluGA044531**	132,792,863	6.09
	1	**Gga_rs13940234**	132,960,547	4.45
	1	Gga_rs13624646	133,345,452	4.31
	17	Gga_rs15792349	8,366,984	4.34
	17	Gga_rs14098115	8,458,039	4.34
	17	Gga_rs14097650	8,891,679	5.42
	26	Gga_rs16203090	3,684,301	5.66
	28	Gga_rs15249217	1,623,905	4.89

^1^ FPD = feather pecks delivered; pEFP = posterior probability of extreme feather pecking; ^2^ Chromosome number; SNPs printed bold are significant in both traits.

**Table 2:** Results of the variance component analyzes.

^1^ Trait	Prevalence	^2^ V_P_ (^3^SE)	^4^ V_A_ (SE)	^5^ H^2^_obs_. (SE)	^6^ H^2^_liab_. (SE)
FPD	−	0.41 (0.03)	0.08 (0.04)	0.20 (0.08)	−
pEFP	0.24	0.15 (0.01)	0.02 (0.01)	0.14 (0.07)	0.26 (0.14)

^1^ For trait abbreviations see Table 1; ^2^ V_p_ = phenotypic variance; ^3^ SE = standard error; ^4^ V_A_ = additive genetic variance; ^5^ h^2^_obs._ = heritability on the observed scale; ^6^ h^2^_liab._ = heritability on the underlying liability scale.

**Table 3:** Top enriched annotation cluster determined with DAVID from genes in significant GWAS region of ^1^ pEFP as well as ^1^ FPD on Chromosome 1.

Category	Term	^2^ p	Genes	^3^ B
Annotation Cluster 1 Enrichment Score 2.22				
REACTOME_PATHWAY	R-GGA-977441	4.8 × 10^−4^	GABRA5, GABRB3, GABRG3	1.3 × 10^−2^
GOTERM_CC_DIRECT	GABA-A receptor complex (GO:1902711)	8.5 × 10^−4^	GABRA5, GABRB3, GABRG3	4.1 × 10^−2^
GOTERM_MF_DIRECT	GABA-A receptor activity (GO:0004890)	9.2 × 10^−4^	GABRA5, GABRB3, GABRG3	3.7 × 10^−2^
INTERPRO	Gamma-aminobutyric acid A receptor (IPR006028)	1.6 × 10^−3^	GABRA5, GABRB3, GABRG3	1.3 × 10^−1^
REACTOME_PATHWAY	R-GGA-975298	1.6 × 10^−3^	GABRA5, GABRB3, GABRG3	2.2 × 10^−2^
INTERPRO	Neurotransmitter-gated ion-channel transmembrane domain (IPR006029)	5.1 × 10^−3^	GABRA5, GABRB3, GABRG3	2.0 × 10^−1^
INTERPRO	Neurotransmitter-gated ion-channel, conserved site (IPR018000)	5.1 × 10^−3^	GABRA5, GABRB3, GABRG3	2.0 × 10^−1^
INTERPRO	Neurotransmitter-gated ion-channel (IPR006201)	5.4 × 10^−3^	GABRA5, GABRB3, GABRG3	1.5 × 10^−1^
INTERPRO	Neurotransmitter-gated ion-channel ligand-binding (IPR006202)	5.4 × 10^−3^	GABRA5, GABRB3, GABRG3	1.5 × 10^−1^
UP_KEYWORDS	Ion channel	6.7 × 10^−3^	CNGA3, GABRA5, GABRB3, GABRG3	3.1 × 10^−1^
UP_KEYWORDS	Synapse	1.9 × 10^−2^	GABRA5, GABRB3, GABRG3	4.1 × 10^−1^
UP_KEYWORDS	Ion transport	2.1 × 10^−2^	CNGA3, GABRA5, GABRB3, GABRG3	3.2 × 10^−1^
GOTERM_CC_DIRECT	cell junction (GO:0030054)	4.5 × 10^−2^	GABRA5, GABRB3, GABRG3	6.7 × 10^−1^
UP_KEYWORDS	Cell junction	5.5 × 10^−2^	GABRA5, GABRB3, GABRG3	5.3 × 10^−1^
UP_KEYWORDS	Cell membrane	9.7 × 10^−2^	GABRA5, GABRB3, GABRG3, RP2	6.7 × 10^−1^

^1^ For trait abbreviations see Table 1; ^2^ p = *p*-value; ^3^ B = Benjamini test to correct for multiple testing.
